# Primary Ion Depletion Kinetics (PIDK) Studies as a New Tool for Investigating Chemical Ionization Fragmentation Reactions with PTR-MS

**DOI:** 10.1371/journal.pone.0066925

**Published:** 2013-06-26

**Authors:** Erna Schuhfried, Tilmann D. Märk, Franco Biasioli

**Affiliations:** 1 Institut für Ionenphysik und Angewandte Physik, Leopold Franzens Universität Innsbruck, Innsbruck, Tyrol, Austria; 2 Food Quality and Nutrition Department, Research and Innovation Centre - Fondazione Edmund Mach, San Michele a/A, Trentino, Italy; Wake Forest University, United States of America

## Abstract

We report on a new approach for studying fragmentation channels in Proton Transfer Reaction-Mass Spectrometry (PTR-MS), which we name primary ion depletion kinetics (PIDK). PTR-MS is a chemical ionization mass spectrometric (CIMS) technique deploying hydronium ions for the chemical ionization. Induced by extremely high concentrations of analyte M, depletion of the primary ions in the drift tube occurs. This is observed as quasi zero concentration of the primary ion H_3_O^+^, and constant MH^+^. Under these non-standard conditions, we find an overall changed fragmentation. We offer two explanations. Either the changed fragmentation pattern is the result of secondary proton transfer reactions. Or, alternatively, the fast depletion of H_3_O^+^ leads to reduced heating of H_3_O^+^ in the drift field, and consequently changed fragmentation following protonation of the analyte M. In any case, we use the observed changes in fragmentation as a successful new approach to fragmentation studies, and term it primary ion depletion kinetics, PIDK. PIDK easily yields an abundance of continuous data points with little deviation, because they are obtained in one experimental run, even for low abundant fragments. This is an advantage over traditional internal kinetic energy variation studies (electric field per number density (*E/N*) variation studies). Also, some interpretation on the underlying fragmentation reaction mechanisms can be gleamed. We measure low occurring fragmentation (<2% of MH^+^) of the compounds dimethyl sulfide, DMS, a compound that reportedly does not fragment, diethyl sulfide DES, and dipropyl sulfide DPS. And we confirm and complement the results with traditional *E/N* studies. Summing up, the new approach of primary ion depletion kinetics allows for the identification of dehydrogenation [MH^+^ -H_2_] and adduct formation (RMH^+^) as low abundant fragmentation channels in monosulfides.

## Introduction

Fragmentation in mass spectrometry has been a long-standing challenge, as it can hamper both, compound identification and quantification. One approach to tackle this is using a soft chemical ionization technique, which greatly reduced or even eliminates fragmentation. Proton Transfer Reaction-Mass Spectrometry, PTR-MS, is the implementation of such a chemical ionization reaction deploying hydronium ions, H_3_O^+^, as primary ion. Chemical ionization via protonation usually yields as most abundant ion the quasi-molecular ion MH^+^. However, frequently some fragmentation still occurs, warranting investigation. On the other side, fragmentation patterns can even give information on a compound, and thus actually help compound identification. In the best case, the underlying pattern is understood for a whole compound class. For example, alcohols are known to undergo water abstraction upon protonation [1], [2] in PTR-MS, yielding a nominal mass of [MH^+^ - 18]. Here, we further investigate organosulfur compounds (sulfides) by conventional variation of the energetic conditions (electric field per particle density (*E/N*) studies) [3] in PTR-MS. Unlike in a related chemical ionization technique, SIFT-MS, selected ion flow tube-mass spectrometry [4], where sulfides do not fragment with H_3_O^+^ [5], in PTR-MS, sulfides display some fragmentation after ionization.: Saturated monosulfides R**–**S**–**R’ undergo fragmentation to the R**–**S^+^ fragment and for most saturated disulfides, R**–**S**–**S**–**R, the most common fragments are R**–**S**–**S^+^ and R**–**S**–**S**–**H_2_^+^ [3], [6]. In the classical approach of *E/N* studies, the ions are accelerated in the electric field of the flow drift tube, and the applied voltages are changed. Here, we develop a new approach, which we call primary ion depletion kinetics, PIDK. It greatly facilitates investigating low abundant (<2%) fragments, and allows for measurements in a quasi-continuous way. With this new approach, we identify previously unknown, low occurring sulfide fragmentation reactions in PTR-MS, namely adduct formation and dehydrogenation. Adduct formation here means that a larger ion is formed that contains a fragment R of the initial molecule M as well as an additional molecule or atoms, e.g. in this case the molecule M, resulting in the adduct MRH^+^. Dehydrogenation concerns the loss of hydrogen. We validate the approach via a simple kinetic simulation and by comparing the identified new reactions to results from conventional *E/N* studies of sulfides.

### i. Highlights:

New approach for studying chemical ionization fragmentation reactions with PTR-MS
Primary ion depletion changes the observed fragmentation
Monosulfides show dehydrogenation at low abundance in PTR-MS
DMS has a fragment at m/z 47, occurring at an abundance below 2% of MH^+^
Dipropyl sulfide shows low occurring but unusual adduct formation in PTR-MS
Adduct formation and dehydrogenation are confirmed via *E/N* studies

## Materials and Methods

### i. Materials

The following compounds were used: dimethyl sulfide (DMS) CAS 75-18-3 from Fluka (99% purity), diethyl sulfide (DES) CAS 352-93-2 from Alfa-Aesar (98% purity), dipropyl sulfide (DPS) CAS 111-47-7 from Aldrich (97% purity).

### ii. Mass Spectrometry

Measurements were performed with a commercially available PTR-MS from Ionicon Analytik GmbH (Innsbruck, Austria) in the HS (High Sensitivity) version equipped with a quadrupole mass analyzer [7]. The PTR-MS was operated under controlled, monitored and constant conditions of drift tube pressure (2.0–2.1 mbar) and the drift tube temperature (60°C), and the inlet was heated to a temperature of 61°C. All signals were corrected for instrumental transmission coefficients, obtained from calibration curve measurements, performed similar as in literature [8], [9]. No correction for background signals was performed.

### iii. Primary Ion Depletion Kinetics (PIDK)

The compound of interest was prepared by adding 0.3 µl of liquid compound to a N_2_ purged vial of 22 ml, and by further diluting the compound-saturated headspace in N_2_ if necessary, usually several times to find the right concentrations of depletion. Due to the handling, the final samples were not in pure N_2_, but rather a combination of N_2_ and some lab-air. The PTR-MS was fed from the vial at its own flux (ca 30 sccm). The vial was replenished with N_2_ from a N_2_ purged glass flask. This results in an approximately logarithmic concentration decrease of the compound in the vial over time. For DMS, DPS, DES, at first, a full mass scan from 21–200 amu was performed with limited depletion, collecting a limited number of measurement points per *m/z* (mass to charge ratio). For DPS, this was complemented by additional mass scans to 240 amu, in order to check for higher ions (and up to 300 amu from a humid sample with a gas stripper, see below). Then, the depletion/replenishment study was performed with selected ions of interest where higher concentrations of fragments were seen. The selection (selected ion mode) was necessary in order to keep measurement times at a minimum in order to preserve the SEM, which was already being used under extreme conditions (>100000 cps) (cps: counts per second). Furthermore, for the primary ion depletion kinetics studies, the typical voltage applied to the drift tube was U_D_ = 600 V, amounting to an *E/N* (electric field per particle density) of about 140 Td [10]. 1 Townsend (1 Td ) corresponds to 10^−17^ V.cm^2^ [11]. We reduced the voltages at lenses US_0_ and US. Thereby the primary ion signal is reduced to ca 3 x 10^6^ cps, about 1/7 of the usual signal, in order to preserve the SEM [8]. For this depletion study, concentrations were chosen in such a way, that over time, a return of the initially depleted primary ion signal at *m/z* 21 to its normal concentration is observed.

Hence, the procedure is basically the same as for transmission curve determination [8], [9]. Attention was paid to the pressures in the drift tube (automatically recorded with the mass range cycles), as upon injection of the compound-N_2_-air mix, usually a short (ca 3 mini-cycles) pressure instability was observed.

### iv. Fragmentation Pattern Studies via *E/N* Variations

PIDK was complemented by fragmentation studies with *E/N* variations performed as described previously [3], by combining *E/N* variation with inert gas stripping.

In brief, a compound-water solution (0.3 µL in 1 L deionized-water) in an inert gas stripper (volume(solution) = 100 mL) made of glass was monitored in full scan mode (*m/z* 21–240 amu) (compare [3], [12], [13]), and with an initial N_2_ flux of typically 40 sccm. The temperature of the water-compound solution was kept constant during a measurement, and varied between measurements from 25 to 27°C. For these, regular values for lenses US_0_ and US were used, and the *E/N* values were varied by changing U_D_ = 400–600 V, which amounts to an *E/N* range from about 90–140 Td [10]. Fragmentation patterns for dehydrogenation were determined for other compounds in a similar manner, for details see [3]. The fragmentation spectra were averaged over 5 cycles per *E/N* value. Usually, fragment ion amounts were averaged by directly calculating the percentages in relation to the protonated primary ion at each cycle and then averaging the percentages from several cycles. At the end of the *E/N* variations, we chose an *E/N* of ∼140 Td and increased the flux. This causes a stronger slope in the time-concentration diagrams of the protonated compound and the corresponding fragments. Thus increasing the flux facilitates fragment identification. At the highest *E/N*, the highest number of fragments is expected, which is why we chose an *E/N* of ∼ 140 Td.

### v. Fragmentation Pattern Analysis

Fragmentation patterns were analyzed with the Elemental Composition calculator [14], by using the elemental composition of C 0–10, H 0–30, S 0–5. Further calculations of the isotope distributions were performed with the Molecular Weight Calculator [15].

## Results and Discussion

### i. General Concept of Primary Ion Depletion Kinetics Studies (PIDK) - Primary Ion Depletion/replenishment Studies

The basic reaction taking place in the drift tube is the protonation of the analyte M with the reagent (primary) ion H_3_O^+^ in order to form a quasi-molecular ion, which can be detected via a detector:
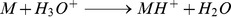
(1)


**Figure 1:**
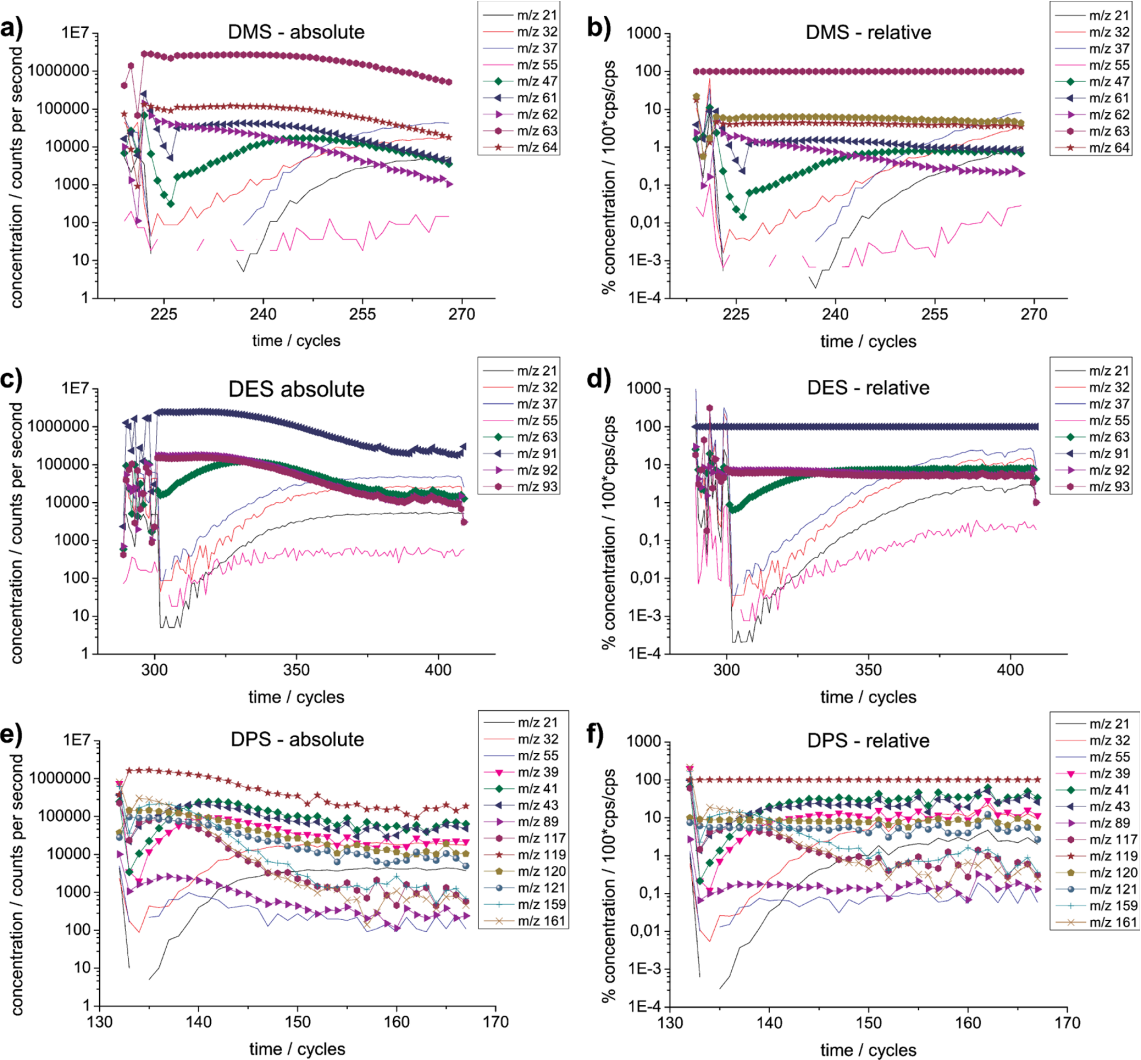
Experimental PIDK results. Primary ion depletion studies of (a), (b) dimethyl sulfide (DMS), (c), (d) diethyl sulfide (DES), (e), (f) dipropyl sulfide (DPS), of absolute (a), (c), (e) and relative (b),(d),(f) concentrations. Relative concentrations are given in percent of MH^+^ (for DMS: *m/z* 63, for DES *m/z* 91, for DPS *m/z* 119). Reagent ions: primary ion *m/z* 19 (monitored via its isotopologue at *m/z* 21), additional primary ion *m/z* 37 and *m/z* 55 and parasitic ion *m/z* 32 are displayed as solid lines for easier distinguishing from reactant ions MH^+^ and fragments, which are displayed as lines+symbol. The time (in cycles) is given from the start of the depletion reaction (and resulting short term instabilities in concentrations) with a maximum depletion as seen in the depletion of primary ion *m/z* 21 until the return to regular non-depleted conditions. Reactant M concentration was changed quasi-logarithmically via a controlled dilution method. Changes in the ion fragmentation abundances are due to primary ion depletion.

The basic principle of the primary ion depletion kinetics studies (and subsequent replenishment) is: Primary ions H_3_O^+^ are depleted by introducing excessive amounts of analyte M into the drift tube. For real examples, see Fig. 1. (We will discuss these figures in detail later on.) Under these “swamping conditions” [16], the amount of reagent ion H_3_O^+^ is limited, which also limits the maximum amount of MH^+^ that can be produced in the protonation reaction (compare Eq. 1). Hence, even when the concentration of M is reduced, here via headspace dilution in a (quasi-) logarithmic manner, the amount of product MH^+^ remains constant. Of course, this holds true not only for H_3_O^+^, but also for the “parasitic” primary ions, H_2_O·H_3_O^+^, (H_2_O)_2_H_3_O^+^, O_2_^+^, NO^+^, respectively, which are depleted in the drift tube by the excessive amounts of analyte M, just as H_3_O^+^ is.

As M is further reduced, less and less primary ions are depleted, and the measured concentrations of primary ions (and parasitic primary ions) return to their usual levels for PTR-MS conditions. Conditions with primary ion depletion are extreme conditions, which are generally avoided in PTR-MS for routine measurements. However, primary ion depletion in PTR-MS has been used for transmission curve determination, e.g. [8], [9]. Albeit, it has been noticed, that this method yields erroneous results when using complete primary ion depletion, and rather incomplete primary ion depletion with lower amounts of M is recommended for obtaining transmission curves [9]. In SIFT, the concentration effects in kinetics is used in a somewhat comparable way for the determination of product ion branching ratios [17], [4].

### ii. Simulation Results with Kintecus

We performed a simple kinetic simulation of the reactions in the drift tube of the PTR-MS with Kintecus [18], see Supplement S1. The drift tube was simulated as plug flow reactor. The concentration of reactant M was logarithmically declining. The simulation clearly shows the two phases of a) depleted primary ion H_3_O^+^ (and its gradual return) and constant product ion MH^+^ and b) a constant primary (reagent) ion and logarithmically declining product ion, as is typical for standard PTR-MS measurements.

### iii. Typical Results of PIDK – Decreased Overall Fragmentation and Occasional Peaks

Concerning the fragmentation, the expected behavior of the fragments would be curves in parallel to MH^+^. Hence, at first a constant concentration is expected and then *log* declining concentration, compare simulation results Supplement S1. However, in the experimental data (Fig. 1), there is clearly a change in the fragmentation pattern. During primary ion depletion and subsequent replenishment, most fragments show an increase of concentration e.g. *m/z* 47 of DMS (Fig. 1a), but some show peaks and diminishing concentrations thereafter e.g. *m/z* 159 of DPS (Fig. 1e). The behavior of the fragment ions cannot be explained by simple reaction kinetics from primary ion depletion. So, why is there a change in fragmentation pattern, when no *E/N* change is imposed externally (e.g. via *U_d_* changes)?

### iv. Interpretation of Depletion Results via Secondary Protonation Reactions

One possible explanation is that advanced secondary ion reactions take place.

The driving force for proton transfer for the protonation reaction (e.g. from H_3_O^+^ to reactant M) is called the change in proton affinity (ΔPA), which is approximately the negative change in Gibbs energy. Under depletion conditions, when there is too much M and too little H_3_O^+^, it could be that the fragment products become reagent ions. For example, with fragments F_1_H^+^ and F_2_, fragment might F_1_H^+^ serve as proton donor:
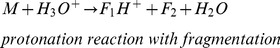
(2)

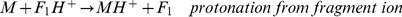
(3)


The precondition is that the reaction has a permissible change in proton affinity, and therefore that the fragment transfers a proton to the reactant M. The neutral fragments F_1_ and F_2_ cannot be monitored in PTR-MS because there is no charge on the molecules. Under regular, non-depleted conditions, the protonation reaction from a fragment is suppressed, because of the predominant concentration of H_3_O^+^ (swamping conditions), and regular protonation from hydronium ions takes place (Eq. 1). Assuming secondary protonation reactions, the observed behavior of the fragment, e.g. *m/z* 47 for DMS, Fig. 1a, becomes clear: Under depletion conditions the concentration of the fragment is lowest, and highest at non-depleted conditions. In the later case it no longer serves as proton donator, consequently no fragment ion is used in secondary reactions and hence the observed concentration of the fragment is highest under non-depleted conditions.

### v. Alternative Interpretation of Depletion Results as Reduced Internal Energy

Alternatively, as second interpretation, we suggest the following mechanism as explanation for the observed fragmentation behavior when going from H_3_O^+^ depleted conditions to non-depleted conditions: A gradual increase of the internal energies of the ions upon gradual replenishment of the primary ion is observed. In particular, under high M concentrations, the primary ions H_3_O^+^ react with the abundant M and are depleted already at the beginning of the drift tube, without the primary ion experiencing average acceleration in the drift tube. Thus less energy is available when the ion-molecule complexes are undergoing the protonation reaction, resulting in less fragmentation. Due to the cooling of the new product ions via buffer gas collisions, the product ions cool immediately after formation. Fragmentation at a later time point would require much more energy, as the full activation energy barrier for dissociation has to be overcome. Overall, the depletion of H_3_O^+^ results in reduced heating of H_3_O^+^ in the drift field, and consequently reduced fragmentation following protonation of the analyte M.

In this case, we can interpret maxima in fragmentation as consequence of the excess concentration of M, in particular for adduct formation and many-body reactions (higher order kinetics which depend non-linearly on the concentration of M).Summing up, by using extremely high concentrations of M, the primary ions H_3_O^+^ (and protonated water clusters and O_2_^+^ and NO^+^) are depleted, which results in a reduced internal energy. Hence, the fragmentation patterns are markedly changed. In such a case clustering reactions and adduct formation are favored, while simple fragmentation reactions are suppressed. Gradual reduction of M results in a gradual increase of the internal energy and a change of fragmentation and fragmentation patterns to normal levels.

### vi. Results from PIDK on Monosulfides

We performed PIDK on 3 monosulfides, the results can be seen in Fig. 1 a,b (DMS) Fig. 1 c,d (DES), Fig. 1 e,f (DPS), given in absolute concentration values in cps (counts per second) (Fig. 1 a,c,e) and in relative values as percent of MH^+^ (Fig. 1 b,d,f). Isotopologues occur when an isotope with a different molecular weight is incorporated in the molecule or ion. The isotopologues for the studied MH^+^ are given in Table 1. Isotopologues are expected to yield perfectly parallel curves. Exceptions to this indicate the presence of an additional fragmentation reaction.

**Table 1:** Isotopologue ratios for protonated sulfides based on theoretical calculations with the Molecular Weight Calculator [15].

protonated compound	protonated mass [amu]	intensity (% of MH^+^)
DMS•H^+^	63.03	100.00
(CH_3_SCH_3_•H^+^)	64.03	3.15
	65.02	4.48
DES•H^+^	91.06	100.00
(CH_3_CH_2_SCH_2_CH_3_•H^+^)	92.06	5.25
	93.05	4.63
DPS•H^+^	119.09	100.00
((CH_3_CH_2_CH_2_)_2_S•H^+^)	120.09	7.46
	121.09	4.76

First we discuss the results for DMS with PIDK. For DMS we discovered 3 fragments with PIDK at *m/z* 61, 62 and 47. To the best of our knowledge, these fragments have not been reported before in the literature, DMS being a compound considered not to fragment. Concerning *m/z* 61, we interpret it as dehydrogenation product [MH^+^-H_2_] from MH^+^ at *m/z* 63.

**Figure 2:**
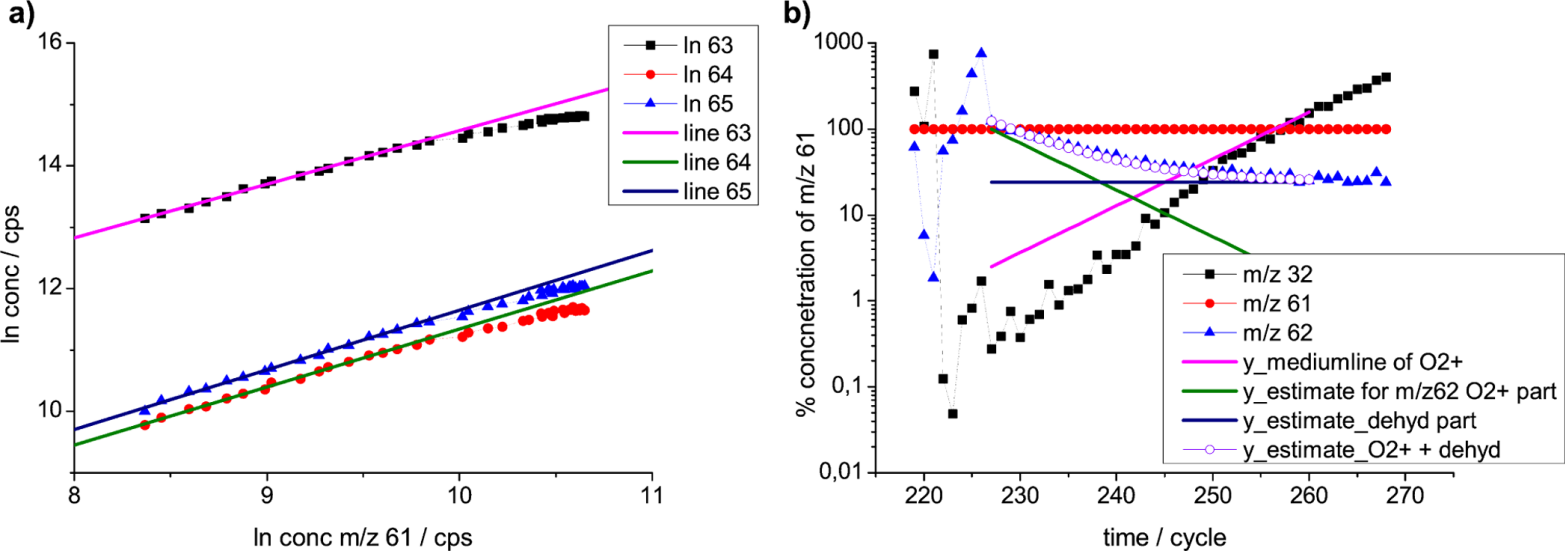
Details for DMS in PIDK. **a) DMS isotopologue ratios.** Demonstration of the deviations from the expected isotopologue ratios. The MH^+^ of DMS is 63, and the isotopolouges are *m/z* 64 and 65. In principle, parallel lines of the isotopologues would be expected, however, this is not perfectly the case. Plot of *log* concentration (conc) *m/z* 61 versus *log* concentration of *m/z* 63, *log m/z* 64 and *log m/z* 65, demonstrating the correct isotopologue ratio and deviation therefrom. Deviations are interpreted as results from dehydrogenation reactions, e.g. *m/z* 63 to *m/z* 61. *m/z* 61 was chosen as x-axis as it is the result solely of dehydrogenation from *m/z* 63 to [MH^+^ - H_2_]. Other signals at various *m/z* are the results of a combination of isotopologues of *m/z* 63 and dehydrogenation reactions. The parallel diagonal lines are intended as help to guide the eye. **b) DMS signal** ***m/z*** **62.** Signal *m/z* 62 (▴) as result of a dehydrogenation from *m/z* 64 and a charge transfer from O_2_ to DMS. The contribution from charge transfer is estimated from the behavior of O_2_^+^ (monitored at *m/z* 32 (▪) (y_mediumline of O2+) as the inverse therefrom (y_estimate for *m/z* 62 O_2_^+^ part) (yielding M^+^). The contribution from the dehydrogenation reaction (yielding [MH^+^ –H_2_] ) to signal *m/z* 62 is estimated from the signal of *m/z* 62 under non-depleted conditions at the end of the monitored time (y_estimate_dehyd part). The two effects together superimpose excellently (○) as y_estimate_O2++dehyd, compared to the measured curve of *m/z* 62 (▴). All concentrations are given as % of *m/z* 61 (•). dehyd = dehydration. dehyd = dehydration.

In order to make the changes from dehydrogenation visible, we plot the dehydrogenation product *m/z* 61 against DMS *m/z* 63 and its isotopologues *m/z* 64 and 65 (Fig. 2a), which makes the isotopologue ratio and effect of dehydrogenation easier discernable. This yields an almost perfect correlation (Fig. 2a), itself confirming dehydrogenation to *m/z* 61. At high concentrations, a small deviation can be seen, which we interpret as additional dehydrogenation from the isotopologues (see guiding lines in Fig. 2a and deviation therefrom). The deviation is hence an additional confirmation of the presence of dehydrogenation [MH^+^-H_2_].

Concerning *m/z* 62, it shows a markedly different behavior compared to *m/z* 61. This can be successfully interpreted as a combination of two effects, namely first, the dehydrogenation as [MH^+^-H_2_] from isotolopologue *m/z* 64, and second, a direct charge transfer via O_2_^+^ from DMS to *m/z* 62, see also Fig. 2b.:
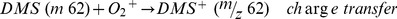
(4)

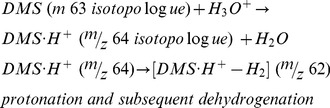
(5)


Firstly, the recovery of the O^2+^ signal (parasitic ion) at *m/z* 32 and, correspondingly, the declining concentration of M^+^ over time as the contribution of the charge transfer reaction from O_2_^+^ from M to M^+^ at *m/z* 62 is estimated. Secondly, the contribution to the signal at *m/z* 62 from the dehydrogenation reaction of MH^+^ to [M -H_2_]^+^ is determined. The combination of these two signals (the estimate being indicated as open circles in Fig. 2b) nicely overlaps with the measured *m/z* 62 signal in Fig. 2b.

Second, we discuss details the behavior of DES in PIDK, which shows a notable decrease in fragmentation. PIDK for DES is depicted in Fig. 1 c and d. The concentration of M is constantly decreased (logarithmically with time). The primary ions deplete (*m/z* 21, 37, 32) (H_3_O^+^ *m/z* 19 is monitored at *m/z* 21), and this results in a constant concentration of reaction products, as can be seen in MH^+^. As the concentration of M falls low enough, the primary ions no longer completely deplete and the reaction product concentration of MH^+^ starts to decrease. The *m/z* 21, 37, 55 concentrations of hydronium ions and parasitic primary ions do not return exactly logarithmically. The reason for this lies in the reaction of m19 to m37 (and m55 etc), for which there is an equilibrium between the protonated water clusters in the drift tube [19]. The known fragment *m/z* 63 of DES is clearly decreased under PIDK conditions and returns to normal concentrations when the depleted primary ions return to non-depleted conditions at the end in the figure (see Fig. 1c and d).

Third, we discuss PIDK of DPS, in particular adduct formation, dehydrogenation, and the occurrence of “peaks” in the abundance of some fragments in PIDK.

**Figure 3:**
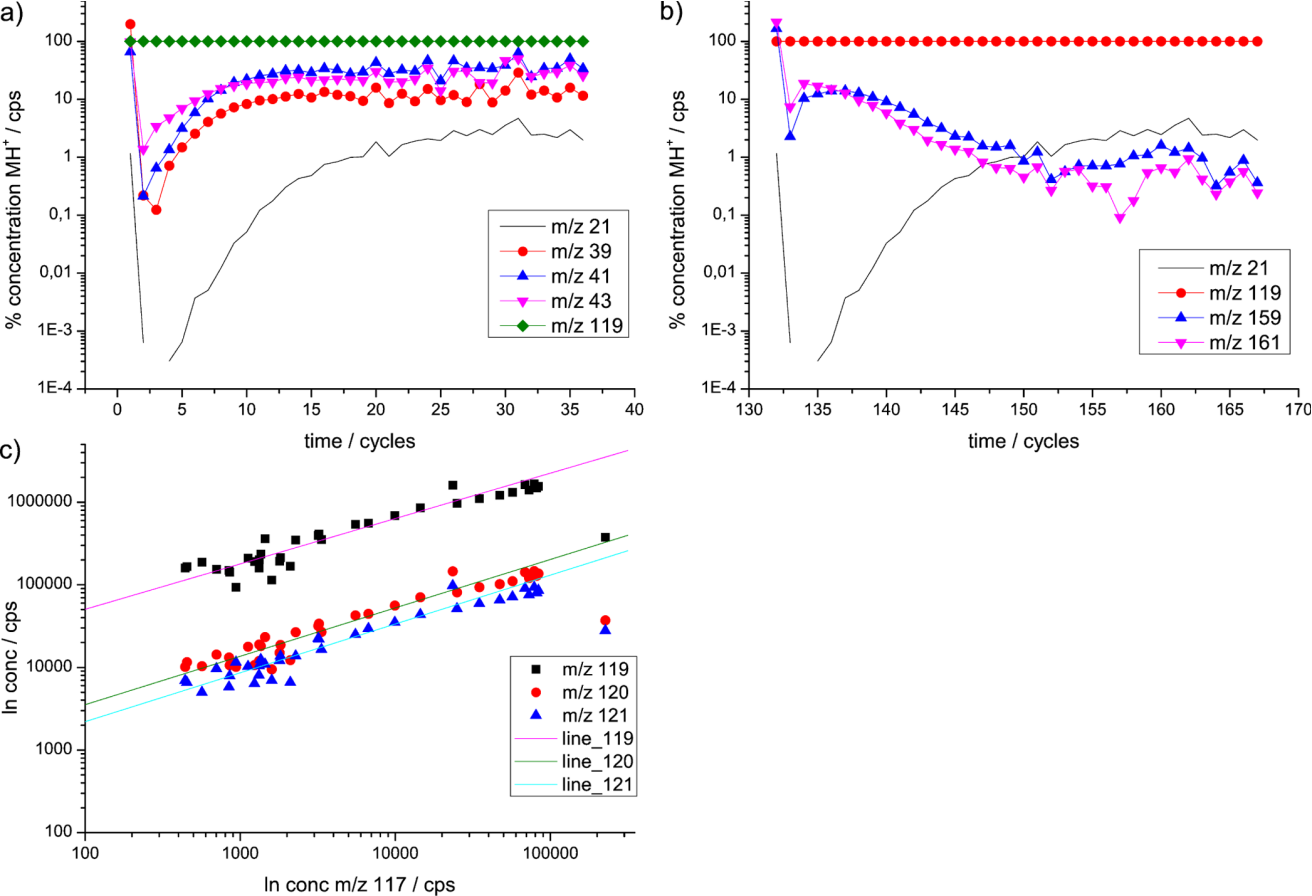
Details for DPS in PIDK. **a) Signals ***m/z* 39, 41 and 43– typically carbon chain fragments.**** Demonstration of the slightly different behavior of *m/z* 43 (▾) compared to *m/z* 39 (•) and 41 (▴) in PIDK. *m/z* 43 is a suspected precursor ion for *m/z* 39 and *m/z* 41, all of which are typically carbon chain fragments. The signals are normalized to 100% MH^+^ at *m/z* 119 (♦). **b) Signals** ***m/z*** **159 and** ***m/z*** **161– typically adduct ions.** Demonstration of the behavior of *m/z* 159 (▴) and *m/z* 161(▾) in PIDK. The signals are normalized to 100% MH^+^ at *m/z* 119 (•). Adduct ions here are supposed to be the result of two (or more) molecule (M) parts. *m/z* 161 and *m/z* 159 probably are R-S-R·R·H^+^ and [R-S-R·R-H_2_]·H^+^, respectively. **c) DMS isotopologue ratios.** Demonstration of the deviations from the expected isotopologue ratios. The MH^+^ of DMS is 63, and the iotopolouges are *m/z* 64 and 65. Plot of *log* concentration (conc) *m/z* 61 versus *log* concentration of *m/z* 63, *log m/z* 64 and *log m/z* 65, demonstrating the correct isotopologue ratio and deviation therefrom because of changes in the ratio. Deviations are explained as results from dehydrogenation reactions. *m/z* 117 was chosen as x-axis as it is the result solely of dehydrogenation from *m/z* 119 to [MH^+^ - H_2_]. Other signals at various *m/z* are the results of a combination of isotopologues of *m/z* 119 and dehydrogenation reactions. The parallel diagonal lines are intended as help to guide the eye.

The results of PIDK studies on DPS can be seen in Fig. 1 e and f, details are depicted in Fig. 3 a-c. DPS displays the typical carbon-chain-fragments (Fig. 3a) such as *m/z* 39, 41 and 43 [2], [20]. Moreover, a dehydrogenation product exists at *m/z* 117, similar to the one seen for DMS (Fig. 1e). Besides that, in addition, clearly adduct formation at *m/z* 159 and 161 takes place (Fig. 3b). We suspect that *m/z* 159 is a dehydrogenation product from *m/z* 161.

Some fragment ions in PIDK show a different behavior with a minimum in concentration under depleted conditions and a peak in the absolute concentration during H_3_O^+^ replenishment, as for example in DPS *m/z* 159 and 161 (Fig. 3b), which are both adduct ions with higher molecular masses than MH^+^. The typical behavior of the fragments in PIDK upon H_3_O^+^ replenishment seems to be an increase in concentration, as can be seen for *m/z* 47 for DMS, *m/z* 63 for DES and *m/z* 39, 41, 43 for DPS.

Following the first interpretation via advanced secondary ion reactions, the reason would be that the smaller fragments have an energetically favorable Gibbs energy for proton transfer to M, compared to water, and correspondingly a favorable ΔPA. This is compatible with the observation that, indeed, smaller molecules tend to have a lower ΔPA (difference in proton affinity) than larger ones. Vice versa, large adduct ions, that are larger than MH^+^, should have a disfavorable ΔPA. Moreover, this interpretation allows for an explanation of the observed behavior of the fragments with a peak maximum during primary ion replenishment: This can be easily explained as a concentration dependency of the probability of the reaction. Considering that the two fragments are adducts, this makes perfectly sense, as at least two species have to interact with H_3_O^+^, e.g. DPS (R-S-R):
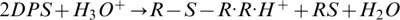
(6)


The necessary two DPS molecules make this reaction second order with respect to DPS, which means the reaction kinetics for fragment *m/z* 161 depends on the quadratic concentration of DPS. This can be seen in the decline in concentration of the adduct when the concentration of DPS declines towards the end of the PIDKs experiment. Moreover, taking a look at the behavior of the concentration of *m/z* 117, it is similar to that of *m/z* 161 and *m/z* 159, which might indicate that it results rather from a ternary reaction of two DPS (or more), than from a direct dehydrogenation reaction of DPS·H^+^.

When applying the alternative interpretation for the behavior of fragment ions in PIDK of a change in internal energy, the following applies:

At higher *E/N*, the elevated collision energy conditions favor rather dissociation than stabilization of higher water cluster hydrates in collisions with the air buffer gas [21]. And the same is true for activated complexes (excited intermediate complexes) involving third bodies, where the reaction rate typically has a “negative energy dependence”, i.e. decrease with increasing energetic conditions [22]. Compare also this reference [10], where the behavior of (H_2_O)_2_ H_3_O^+^ at *m/z* 55 compared to the behavior of protonated acetone at *m/z* 59 in an *E/N* study was interpreted as ternary versus binary reaction. A similar effect is seen in the *E/N* dependence of higher protonated water clusters [19]. We take this increased sensitivity of cluster stability on the energetic conditions, compared to non-clusters, and conclude that the rapid decrease of relative (to MH^+^) fragment abundance of *m/z* 161 (and of *m/z* 159) is the result of *m/z* 161 being a weakly bond cluster – indeed, an adduct ion (which it has to be because of the higher mass than MH^+^). This could be seen as an additional confirmation of the interpretation of *m/z* 161 as adduct ion. Moreover, this seemingly confirms that PIDK conditions favor clusters, the formation of adduct ions and the formation of ternary complexes, similar as would lower *E/N* conditions.

Regardless which interpretation is correct, both would explain the two behaviors of fragment ions of either increased concentrations in primary ion replenishment or the appearance of peaks.

### vii. *E/N* Studies

Because of the similarities between PIDK and reduced *E/N* conditions, we compare our results from PIDK to results from *E/N* studies. We rechecked if we could find the newly identified fragments from PIDK studies also in *E/N* studies. For *E/N* studies, these fragments are difficult to distinguish from the background due to the low concentrations at which they are found, and hence difficult to identify. On the other hand, *E/N* studies allow for confirming fragments when combined with inert gas stripping: Therein a logarithmic decrease of the volatile compound in the gas phase is achieved. In the mass spectrometer, any fragment derived from the volatile compound has to show the same decrease of concentration as the protonated parent ion. Hence true fragments have to show a slope identical to the parent ion (compare [6], [3]).

The results from the *E/N* studies are tabularized in Table S2_1 in Supplement S2. We specifically looked for MH^+^-H_2_ fragments, MRH^+^ fragments and other adducts with a *m/z* higher than MH^+^ (arranged in three groups in Table S2_1 in Supplement S2). Keep in mind that due to the low concentrations involved (in *log* scale), no background corrections were performed and the values in Table S2_1 in Supplement S2 present maximum values, e.g. for the fragment of DPS, which would be below the concentration of 2% of MH^+^ when corrected for background.

### viii. *E/N* Study on DMS

**Figure 4:**
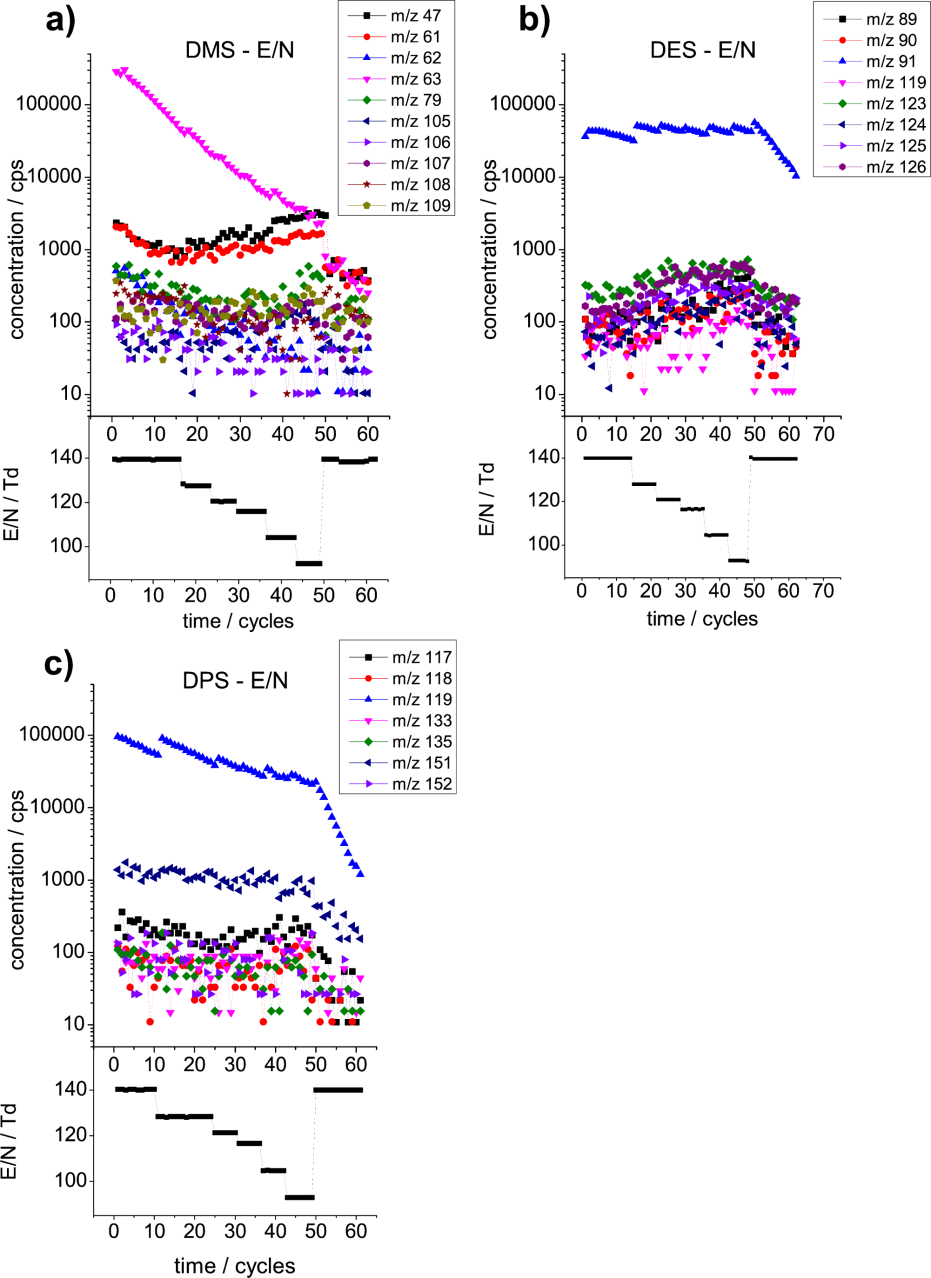
*E/N* variation study for minor fragment pathways. Studied for (a) DMS (b) DES (c) DPS, dehydrogenation and adduct ion study. MH^+^ is for (a) DMS: *m/z* 63, (b) DES: *m/z* 91, (c) DPS: *m/z* 119. The *E/N* values at each measurement point are displayed below each plot for the resulting ions. The slopes in *log* scale are from inert gas stripping for an *E/N* range varied from ∼90–140 The parallel slopes confirm that the fragments are true fragments from the measured sulfides, and hence this proves the existence of dehydrogenation and adduct formation in mono-sulfides. No background correction was performed.

MH^+^ of DMS is *m/z* 63. The fragment at *m/z* 47, which we first found by PIDK, is confirmed via traditional *E/N* studies (see Fig. 4 a). Moreover, *E/N* studies find a number of adduct ions, namely *m/z* 79, 105–109 (see Fig. 4 a and Table S2_1 in Supplement S2). In addition, the dehydrogenation products at *m/z* 61 and 62 for DMS are confirmed (see Fig. 4a). The DMS *E/N* study shows that the studied fragments in Fig. 4a are affected by background signals, as seen by the less steep slope compared to the signal for MH^+^ at *m/z* 63. Moreover, *m/z* 47, 61, 79 are strongly influenced by isotopologues of protonated water cluster ions, such as H_2_O·H_3_O^+^ for *m/z* 47, (H_2_O)_2_H_3_O^+^ for *m/z* 61, (H_2_O)_3_H_3_O^+^ for *m/z* 79. Fragments *m/z* 105 and 108 indicate that the actually relevant ions are rather at odd *m/z* ratios. However, the lack of a clear slope seen at the corresponding odd *m/z* ratio ions (*m/z* 105, 107, 109) indicate overlapping ion signals there, thus concealing their relevancy. For ion *m/z* 105, only for the highest *E/N* (ca 140 Td), some slope is seen.

In SIFT-MS, a related technique to PTR-MS using preselected primary ions, for H_3_O^+^ as reagent ion, the product adduct ions MH^+^H_2_O are reported for DMS [23]. Moreover, the authors report as reaction products for adduct ions of dry air with 5% CO_2_ the adduct ions H_3_O^+^N_2_ (*m/z* 47) and H_3_O^+^CO_2_ (*m/z* 63) [23]. In principle, these two ions could concern DMS in PTR-MS. However, because of the existing slope we can confirm that *m/z* 47 is a true fragment of DMS (but there might be some additional H_3_O^+^N_2_ causing and explaining the strong E/N dependence at low E/N). DMS can be monitored at *m/z* 63, however, for this no clear deviation of the slope due to a H_3_O^+^CO_2_ adduct ion is seen. Hence it is rather unlikely that considerable amounts of H_3_O^+^CO_2_ are present. Mind that *m/z* 109 overlaps with a higher protonated water cluster.

### ix. E/N Study Details on DES

The *E/N* study of DES finds ion products at *m/z* 89 and 90 (Fig. 4b), consistent with another dehydrogenation product [MH^+^-H_2_] from MH^+^ and of an isotopologue of MH^+^. Because of the low threshold concentration, we only found this dehydrogenation product when specifically looking for it. Moreover, adduct ions are found and confirmed via their slope (see Fig. 4b), at *m/z* 119, 123–126, respectively.

### x. *E/N* Study Details on DPS

The E/N study for DPS again confirms the existence of dehydrogenation products [MH^+^-H_2_] at *m/z* 117 and 118, see Fig. 4c. Adduct ions are found at *m/z* 133, 135, 151 and 152. Unfortunately, the adduct ions 159 and 161 could not be confirmed due to the low concentrations at which they were found.

### xi. Discussion of PIDK

So far, nothing comparable to PIDK has been reported. PIDK allows for the identification of fragment ions that else would be overlooked. And PIDK allows for distinguishing three groups of ions: 1) isotopologues that have a parallel behavior to the protonated quasi-molecular parent ion, 2) fragments that show a minimum concentration under depletion conditions 3) fragments (seemingly adduct ions) that have a peak in concentration during replenishment of the primary ion H_3_O^+^. PIDK has several advantages over traditional *E/N* studies. It is fast once the right concentrations are found, and it yields lots of continuous data points with little deviation. Neither adduct formation, nor dehydrogenation have been reported so far for sulfides, neither in our work with PTR-MS [3], nor in related SIFT studies [5] [24] [25]. With PIDK it was possible to identify dehydrogenation and adduct formation as minor fragmentation reactions of sulfides, which are not easily found in *E/N* studies, and here were identified only retrospectively after finding them in PIDK and looking for them in a targeted way in an *E/N* study. Moreover, it might be possible to draw conclusions on the underlying reaction mechanisms. It seems that more complex (more body reactions) reactions seem to have an increased abundance in PIDK. In principle, it might be possible to make a statement on the energetic barriers of the fragmentation reactions by considering which fragmentation reaction peaks first, when also knowing the reaction order.

PIDK also gives the first explanation for the deviation of transmission curves when obtained from completely depleted ion conditions [9]: Probably else irrelevant fragmentation reactions become relevant (adduct formation), and the concentration of the monitored ion is seen as too low, hence resulting in too high transmission coefficients for the corresponding *m/z*.

However, PIDK are not easy to interpret and the influence of other molecules, such as H_2_O or CO_2_, cannot be ruled out when depicting adduct formation reactions. *E/N* studies can serve to confirm the found fragments when performed in combination with inert gas stripping, as was done here.

### Conclusions

We report a new approach for studying fragmentation channels in Proton Transfer Reaction-Mass Spectrometry (PTR-MS). PTR-MS is a chemical ionization mass spectrometry (CIMS) technique deploying hydronium ions for the chemical ionization. Induced by extremely high concentrations of analyte M, depletion of the primary ion occurs. Upon depletion and gradual replenishment of primary ions via reduction of the analyte M, an unexpected behavior in the concentration of fragments can be observed. The changed fragmentation patterns mostly consist of less fragmentation than expected, however, also peaks in the concentration of fragments are observed. As mechanism for the changed fragmentation we suggest two explanations. The first involves advanced secondary ion reactions. The second postulates a change in the internal ion energies. As examples we present dimethyl sulfide, DMS, a compound that reportedly does not fragment, diethyl sulfide DES, and dipropyl sulfide. The new approach easily yields an abundance of consistent data, because obtained in one run, a big advantage over traditional *E/N* variation studies. Thus it was possible to identify a low abundance fragmentation channel: At below 2% of total fragmentation, dimethyl sulfide and DPS upon protonation show dehydrogenation to [MH^+^ -H_2_]. Furthermore, we present supporting quantitative data from traditional *E/N* studies of low occurring fragments below 2% of MH^+^ abundance. This demonstrates that dehydrogenation is a low occurring, however, common reaction channel in monosulfides. For dipropyl sulfide (R**–**S**–**R, molecular mass 118), we identify with primary ion depletion kinetics a hence unknown adduct formation, yielding an *m/z* 161 ion (RMH^+^), and an ion at *m/z* 159. Data from traditional *E/N* fragmentation studies confirm the presence of low occurring (<2% of MH^+^ abundance) adduct fragments in all monosulfide studied. Summing up, the new approach of primary ion depletion kinetics allowed for the identification of dehydrogenation and adduct formation as fragmentation channels in mono-sulfides. We hope that the new technique of PIDK will prove useful for future studies of reaction channels.

## Supporting Information

Supplement S1 **Kinetic simulation of primary ion depletion.**
(PDF)

Supplement S2 ***E/N*** **study.**
(PDF)
