# Supplementary material for: Primary Ion Depletion Kinetics (PIDK) Studies as a New Tool for Investigating Chemical Ionization Fragmentation Reactions with PTR-MS
Source: PLoS One. 2013 Jun 26;8(6):e66925. doi: 10.1371/journal.pone.0066925 (PMC3694147; doi:10.1371/journal.pone.0066925)
Supplement: Supplement S1 — Kinetic simulation of primary ion depletion. (PDF) [file pone.0066925.s001.pdf]

Supplement S1

Kinetic simulation of primary ion depletion

In order to better understand the reactions taking place under depletion conditions, we performed a simulation of the kinetic reactions.

a) Literature for kinetic simulations for PTR-MS

For that reason, we investigated the underlying theory of the reactions. For the related SIFT tube reactions, a general theory can be found here [1], [2]. A general derivation of reaction kinetics in the drift tube can be found e.g. here [3]. For the PTR-MS system, Vlasenko et al [4] and Inomata et al [5] applied first principle considerations of drift tube kinetics for deriving the humidity dependence of the protonation reaction of formaldehyde in the drift tube. Interestingly, Valsenko et al [4] found a reaction time dependence of the protonation products and dependence on the water content due to not-reaching equilibrium. In SIFT, the reactions of water and water clusters have been modeled for calculating reaction constants.

b) Simulation with Kintecus

The reactions in the flow-drift tube of the PTR-MS were simulated with Kinetcus [6]. The simulation assumes that the drift tube is s plug flow reactor, which assumes the reactions are taking place in a special slices (plugs) which travel along the reaction tube and with a limited residence time, and independent from the other plugs (in order to simulate a plug flow reactor rather than a continuously stirred reactor CSTR). The residence time was assumed to be 0.1s. The studied reactions are given in Table_S1_1. For details of the reaction conditions see Table_S1_2

A logarithmically declining concentration of B was programmed. B reacts with the primary ion H_3O^+:

$$B + H_3O^+ \text{->} BH^+ + H_2O \qquad \text{Eq. S1\_1}$$

Also the behavior of the reaction with the parasitic primary ion O_2^+ is given:

$B + O_2^+ \rightarrow B^+ + O_2$ Eq. S1_2

Moreover, fragmentation reactions of BH^+ to F^+ and I^+ and subsequent fragmentation of I^+ to K^+ are considered.

The simulation results clearly show two phases, see Fig. S1_1: The depletion stage, at which primary ion is depleted and causes a constant concentration in BH^+, even though the concentration of B is logarithmically declining. The second phase is the non-depleted stage, in which a logarithmic decline in B is seen as logarithmic decline in BH^+. The latter phase represents the typical measurement conditions in PTR-MS.

Fig. S1_1

Kintecus simple simulation of primary ion depletion

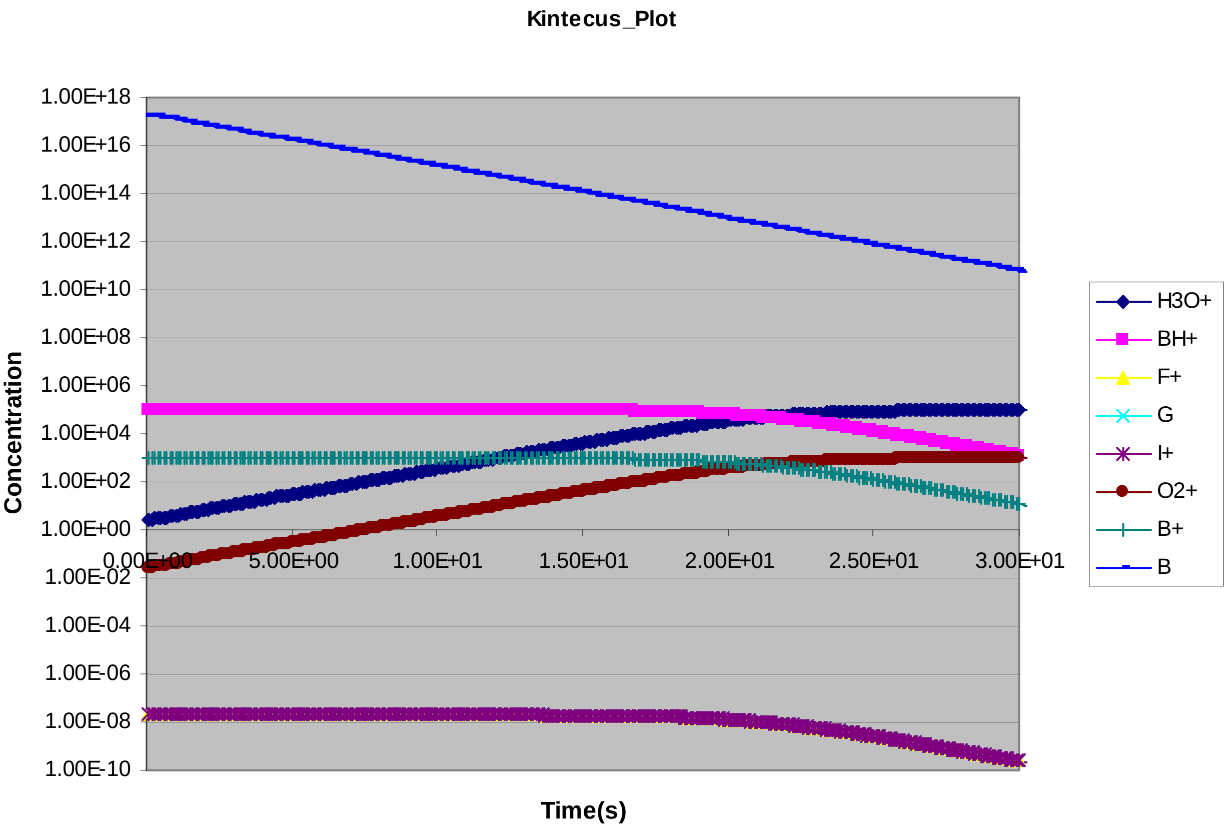

Table S1_1
Kintecus reactions and reaction
as programmed in Kintecus. The units for k are cm3/sec per molecule.

reaction rate	chemical reaction
2.00E-09	B + H3O+ ==> BH+ + H2O
#0.00000000001	
#0.1	
#1	
2.00E-09	BH+ ==> F+ + G
2.00E-09	BH+ ==> I+ + H2
#0.000000002	I+ ==> K+ + H2
2.00E-09	B + O2+ ==> B+ + O2
2.00E-09	B ==> P
#0.000000001	B + O2+ ==> B+ + O2

#0.000000000001 B ==> P
do not remove this END
do not remove this END
do not remove this END
END

Table S1_2
Details of reaction conditions for the kintecus simulation
Alog 11.txt is a file containting a continously logarithmically declining concentration for B.

#	Time in CSTR(s)	Conc.	(Y/N) ?	Conc.	(Y/N)	(Filename/#/No)	Comments
**B**	**1.00E-04**	**0**	**y**	**0**	**No**	Alog11.txt	Alog11.txt
H3O+	**1.00E-04**	0.000	**y**	1.00E+05	No	No	1.00E+05
**BH+**	**1.00E-04**	**0.000**	**y**	**0**	**No**	No	Alog14.txt for log model or Alog16.txt
H2O	**1.00E-04**	0.000	**n**	0	No	No	0
F+	**1.00E-04**	0.000	**y**	0	No	No	0
G	**1.00E-04**	0.000	**y**	0	No	No	0
I+	**1.00E-04**	0	**y**	0	No	No	0
H2	**1.00E-04**	0.00E+00	**n**	0	No	No	0
O2+	**1.00E-04**	0	**y**	1.00E+03	No	No	0
B+	**1.00E-04**	0	**y**	0.00E+00	No	No	1.00E+03
O2	**1.00E-04**	0	**n**	0	No	No	1.00E+01
P	**1.00E-04**	0	**n**	0	No	No	1
END							

Literature

[1] L.A. Viehland, S.L. Lin, E.A. Mason, Kinetic theory of drift-tube experiments with polyatomic species, Chemical Physics. Chemical Physics 54 (1981) 341–364.
[2] K. Kumar, The physics of swarms and some basic questions of kinetic theory, Physics Reports. Physics Reports 112 (1984) 319–375.
[3] S.-B. Woo, J.H. Whealton, Transport Model for Converting Charged Species in Drift Tubes, Phys. Rev. Phys. Rev. 180 (1969) 314.
[4] A. Vlasenko, A.M. Macdonald, S.J. Sjostedt, J.P.D. Abbatt, Formaldehyde measurements by Proton Transfer Reaction – Mass Spectrometry (PTR-MS): correction for humidity effects, Atmos. Meas. Tech. Discuss. Atmos. Meas. Tech. Discuss. 3 (2010) 965–988.
[5] S. Inomata, H. Tanimoto, S. Kameyama, U. Tsunogai, H. Irie, Y. Kanaya, u. a., Technical Note: Determination of formaldehyde mixing ratios in air with PTR-MS: laboratory experiments and field measurements, Atmos. Chem. Phys. Atmos. Chem. Phys. 8 (2008) 273–284.
[6] J.C. Ianni, Kintecus, 2011.
