# Supplementary material for: Primary Ion Depletion Kinetics (PIDK) Studies as a New Tool for Investigating Chemical Ionization Fragmentation Reactions with PTR-MS
Source: PLoS One. 2013 Jun 26;8(6):e66925. doi: 10.1371/journal.pone.0066925 (PMC3694147; doi:10.1371/journal.pone.0066925)
Supplement: Supplement S2 — E/N study. (PDF) [file pone.0066925.s002.pdf]

Supplement S2

E/N study

of the fragmentation pattern of low occurring Mono-sulfide fragments (dehydrogenation fragments and large adduct ions)

The E/N range is ~90-140 Td.

The ions are grouped in 3 groups for each sulfide for

1) dehydrogenation products, e.g. MH^+ -2H

2) adducts, such as of MRH^+, MRH^+-H_2, MRH_2+H^+ and

3) actually identified most abundant adduct ions at E/N ~ 140 Td, confirmed by their slope.

Ions confirmed as fragments or adduct ions via their correct slopes in inert gas stripping are given in bold.

Table S2_1

(a) Dimethylsulfide CH_3SCH_3 (MW: 62)

exact E/N [Td]	**product ions (m/z / Th) ***	**% of MH^+** *92*	**% of MH^+** *104*	**% of MH^+** *116*	**% of MH^+** *121*	**% of MH^+** *128*	**% of MH^+** *140*	**interpreted ion (tentative)**
	60	4.41	2.73	1.07	0.51	0.23	0.06	M^+ - 2H
	**61**	54.81	26.86	12.09	5.97	2.59	0.80	**MH^+ - H_2**
	**62**	1.53	0.97	0.81	0.53	0.39	0.19	**MH^+ - H_2 isotopologue; M^+ (via CT)**
	**63**	100.00	100.00	100.00	100.00	100.00	100.00	MH^+ (R-S-R)
	75[d]	14.31	4.25	1.96	0.87	0.24	0.06	
	76[d]	1.05	0.22	0.07	0.03	0.00	0.00	MRH^+ -2H
	77[d]	11.26	2.81	0.52	0.22	0.08	0.03	MR^+
	78[d]	0.68	0.13	0.06	0.05	0.01	0.01	MRH^+
	**79[EN]**	14.98	4.02	2.32	0.89	0.68	0.25	$MRH.H^+$
	80[d]	0.02	0.05	0.18	0.20	0.51	1.30	$MRH_2.H^+$
	**105**	3.85	2.04	0.82	0.37	0.12	0.04	$C_3H_5S_2$; $C_5H_{13}S$; C_6HS; C_8H_9;
	**106***	0.64	0.57	0.26	0.19	0.14	0.04	
	**107**	5.52	2.60	1.21	0.79	0.42	0.10	$C_3H_7S_2$; C_6H_3S; C_8H_{11};
	**108***	2.51	1.14	1.06	0.60	0.43	0.15	
	**109**	7.40	3.19	2.14	1.01	0.36	0.09	CHS_3; $C_3H_9S_2$; C_6H_5S; C_9H; W_5
	**47[f]**	101.23	50.25	18.75	9.36	3.41	0.87	

(b) Diethyl sulfide $CH_3CH_2SCH_2CH_3$ (MW: 90)

	**product ions (m/z / Th) ***	**% of MH^+**	**% of MH^+**	**% of MH^+**	**% of MH^+**	**% of MH^+**	**% of MH^+**	**interpreted ion (tentative)**
exact E/N [Td]		***93***	***105***	***116***	***121***	***128***	***140***	
	88	0.09	0.10	0.05	0.05	0.04	0.01	M^+ - 2H
	**89**	0.76	0.60	0.34	0.31	0.29	0.22	**MH^+ - H_2**
	**90**	0.53	0.38	0.30	0.30	0.19	0.18	**MH^+ - H_2 isotopologue; M^+ (via CT)**
	**91**	100.00	100.00	100.00	100.00	100.00	100.00	MH^+ (R-S-R)
	118u	0.01	0.04	0.01	0.03	0.01	0.01	MRH^+ -2H
	**119**	0.27	0.19	0.15	0.08	0.09	0.18	MR^+
	120u	0.16	0.26	0.25	0.28	0.23	0.20	MRH^+
	121u	0.18	0.17	0.09	0.11	0.07	0.09	$MRH\cdot H^+$
	122u	0.05	0.06	0.07	0.05	0.03	0.04	$MRH_2\cdot H^+$
	**123**	1.28	1.20	1.10	1.07	0.84	0.55	$MRH_3\cdot H^+$
	**124**	0.37	0.37	0.34	0.31	0.24	0.11	
	101u	0.63	0.59	0.45	0.34	0.24	0.16	
	107u	0.74	0.71	0.61	0.52	0.64	0.79	
	108u	0.18	0.24	0.30	0.26	0.39	0.71	
	109[w6]	0.41	0.49	0.41	0.33	0.26	0.13	
	**123**	see above						$C_2H_3S_3$; $C_4H_{11}S_2$;C_7H_7S; C_9H_{15}; $C_{10}H_3$;
	**125**	0.61	0.61	0.72	0.41	0.34	0.26	$C_2H_5S_3$; C_5HS_2; C_7H_9S; C_9H_{17}; $C_{10}H_5$;
	**126**	1.29	1.12	1.05	0.88	0.53	0.31	$C_2H_6S_3$; $C_5H_2S_2$; $C_7H_{10}S$; C_9H_{18}; $C_{10}H_6$;

(c) Dipropyl sulfide $(CH_3CH_2CH_2)_2S$ (MW: 118)

	**product ions (m/z / Th) ***	**% of MH^+**	**% of MH^+**	**% of MH^+**	**% of MH^+**	**% of MH^+**	**% of MH^+**	**interpreted ion (tentative)**
exact E/N [Td]		***93***	***105***	***117***	***121***	***128***	***140***	
	116^t	0.09	0.09	0.04	0.01	0.00	0.01	M^+ - 2H
	**117**	0.90	0.72	0.52	0.36	0.27	0.31	**MH^+ - H_2**
	**118**	0.38	0.26	0.13	0.16	0.10	0.10	**MH^+ - H_2 isotopologue; M^+ (via CT)**
	**119**	100.00	100.00	100.00	100.00	100.00	100.00	MH^+ (R-S-R)
	160^n	0.00	0.00	0.00	0.02	0.00	0.04	MRH^+ -2H
	161^n	see below						MR^+
	162^n	0.03	0.00	0.00	0.00	0.00	0.01	MRH^+
	163^n	0.18	0.03	0.02	0.04	0.06	0.01	$MRH.H^+$
	164^n	0.00	0.00	0.00	0.00	0.00	0.00	$MRH_2.H^+$
	165^n	0.13	0.08	0.04	0.00	0.05	0.03	$MRH_3.H^+$
	166^n	0.00	0.00	0.00	0.00	0.01	0.01	
	**133**	0.48	0.36	0.23	0.12	0.14	0.09	
	134	0.12	0.06	0.05	0.03	0.01	0.02	
	**135**	0.21	0.16	0.13	0.16	0.12	0.11	
	136	0.09	0.09	0.04	0.03	0.01	0.02	
	137	0.31	0.22	0.13	0.08	0.06	0.03	
	143u	0.83	0.48	0.18	0.13	0.05	0.03	
	144	0.08	0.06	0.06	0.02	0.02	0.01	
	145	0.18	0.07	0.05	0.04	0.03	0.01	
	**151**	3.19	3.00	2.94	2.24	2.18	1.77	$C_4H_7S_3$; $C_6H_{15}S_2$;$C_7H_3S_2$;$C_9H_{11}S$; $C_{11}H_{19}$; $C_{12}H_7$;
	**152**	0.16	0.27	0.17	0.23	0.14	0.10	
	153u	0.38	0.24	0.16	0.21	0.18	0.07	
	154	0.00	0.02	0.00	0.00	0.00	0.01	
	155	0.25	0.14	0.02	0.03	0.05	0.02	
	159u	0.22	0.05	0.04	0.05	0.04	0.15	
	160	0.00	0.00	0.00	0.02	0.00	0.04	
	161u	0.06	0.03	0.06	0.02	0.03	0.09	

Legend:

CT charge transfer (from O_2^+)

ions in bold italics are the ions of interest

191 identified product ions are printed in bold; ions not in bold (not considered ions) are used to calculate isotopologue ratios in order to help identify the ions.

u slope not convincing, thus unclear if true fragment/adduct of the sulfide

f confirmation of the identified fragment via IGS

d slope seen but in doubt because overlap with water cluster ions

CT charge transfer

EN new fragment identified via E/N variation

MW Molecular Weight

* unusual even m/z number, indicating an overlap of ions of different origin at the odd m/z, thus concealing a possible slope

W4 protonated watercluster $(H_2O)_4 \cdot H_2O \cdot H^+$ at m/z 91

W_5 protonated watercluster $(H_2O)_5 \cdot H_2O \cdot H^+$ at m/z 109

t signal too low to judge if slope present

n no slope detected/signal too low to judge

w1 protonated watercluster at m/z 37

w2 protonated watercluster at m/z 55

w3 protonated watercluster at m/z 73

w4 protonated watercluster at m/z 91

w5 protonated watercluster at m/z 109

w6 protonated watercluster at m/z 127

w7 protonated watercluster at m/z 145

w8 protonated watercluster at m/z 163

w9 protonated watercluster at m/z 181

w10 protonated watercluster at m/z 199
